# Reprogramming of lysosomal gene expression by interleukin-4 and Stat6

**DOI:** 10.1186/1471-2164-14-853

**Published:** 2013-12-05

**Authors:** Louise M Brignull, Zsolt Czimmerer, Hafida Saidi, Bence Daniel, Izabel Villela, Nathan W Bartlett, Sebastian L Johnston, Lisiane B Meira, Laszlo Nagy, Axel Nohturfft

**Affiliations:** 1 Division of Biomedical Sciences, Molecular and Metabolic Signaling Centre, St. George’s University of London, Cranmer Terrace, London SW17 0RE, UK; 2 Department of Biochemistry and Molecular Biology, Research Center for Molecular Medicine, University of Debrecen, Medical and Health Science Center, Egyetem tér 1, Debrecen H-4010, Hungary; 3 Faculty of Science, Engineering and Computing, School of Pharmacy and Chemistry, Kingston University, Kingston Upon Thames KT1 2EE, UK; 4 Faculty of Health and Medical Sciences, University of Surrey, Guildford GU2 7XH, UK; 5 Instituto Royal, Av. Bento Gonçalves, 9500 Porto Alegre, RS, Brazil; 6 MRC & Asthma UK Centre in Allergic Mechanisms of Asthma, Airway Disease Infection Section, National Heart and Lung Institute, Imperial College London, Norfolk Place, London W2 1PG, UK; 7 MTA-DE “Lendulet” Immunogenomics Research Group, University of Debrecen, Debrecen H-4012, Hungary

## Abstract

**Background:**

Lysosomes play important roles in multiple aspects of physiology, but the problem of how the transcription of lysosomal genes is coordinated remains incompletely understood. The goal of this study was to illuminate the physiological contexts in which lysosomal genes are coordinately regulated and to identify transcription factors involved in this control.

**Results:**

As transcription factors and their target genes are often co-regulated, we performed meta-analyses of array-based expression data to identify regulators whose mRNA profiles are highly correlated with those of a core set of lysosomal genes. Among the ~50 transcription factors that rank highest by this measure, 65% are involved in differentiation or development, and 22% have been implicated in interferon signaling. The most strongly correlated candidate was Stat6, a factor commonly activated by interleukin-4 (IL-4) or IL-13. Publicly available chromatin immunoprecipitation (ChIP) data from alternatively activated mouse macrophages show that lysosomal genes are overrepresented among Stat6-bound targets. Quantification of RNA from wild-type and Stat6-deficient cells indicates that Stat6 promotes the expression of over 100 lysosomal genes, including hydrolases, subunits of the vacuolar H^+^ ATPase and trafficking factors. While IL-4 inhibits and activates different sets of lysosomal genes, Stat6 mediates only the activating effects of IL-4, by promoting increased expression and by neutralizing undefined inhibitory signals induced by IL-4.

**Conclusions:**

The current data establish Stat6 as a broadly acting regulator of lysosomal gene expression in mouse macrophages. Other regulators whose expression correlates with lysosomal genes suggest that lysosome function is frequently re-programmed during differentiation, development and interferon signaling.

## Background

Cells must be able to flexibly adjust the structural and functional capacity of their compartments in order to adapt to stress or changing nutrients, to assume specialized tissue functions and to maintain homeostasis.

The biogenesis of cellular organelles involves the assembly and targeting of numerous proteins and membrane lipids, and often these processes are orchestrated by transcription factors whose activities are adjusted in response to stress or developmental cues. While much is known regarding the regulation of lipids, mitochondria, peroxisomes and the ER [1-6], understanding the transcriptional regulation of lysosomal function remains less advanced.

Lysosomes are defined by acidic luminal pH, characteristic membrane proteins and lipids, and the presence of multiple acidic hydrolases that catalyze the degradation of material reaching the compartment through fluid-phase endocytosis, phagocytosis or autophagy [7-10]. Abnormalities of lysosomal function, content, number, morphology or gene expression are characteristic of multiple inherited lysosomal storage diseases, of cellular senescence, organismal ageing, atherosclerosis, Alzheimer’s and other neurodegenerative diseases [11-17]. Ectopic secretion of lysosomal proteases can lead to excessive extracellular matrix degradation, which in turn contributes to metastasis, emphysema, atherosclerosis, arthritis, osteoporosis and the formation of aneurysms [14,18-20].

Large-scale gene expression correlation analyses have shown that a number of lysosomal genes form coordinated clusters, or synexpression groups, suggesting that expression of these targets is co-regulated under varying conditions [21-23]. Sardiello et al. performed a pattern search of lysosomal promoters, leading to the identification of a specific E-box, which was found to be recognized by a basic helix-loop-helix transcription factor called TFEB [21,23]. Ectopically expressed TFEB causes an upregulation of multiple lysosomal genes, leading to increased numbers of lysosomes, enhanced degradation of endocytic substrates, and lysosomal exocytosis [21,24].

Transcriptional regulation of lysosomal function has been studied mainly during autophagy, and in this context several transcription factors have been shown to play roles in lysosomal gene regulation, including GATA-1 [25], FoxO3 [25] and TFEB [26-29].

Lysosomal substrates of extracellular origin impose a particular load on macrophages and other phagocytic myeloid cells that process microbes, senescent cells and effete tissue material [11,30]. How the degradative capacity of lysosomes in such cells is regulated during stress and differentiation remains poorly understood.

Here, we used expression correlation analyses to search for novel regulators of lysosome-specific genes. We found that transcription factors whose expression correlates with lysosomal genes are often involved in differentiation, embryonic development and interferon signaling. The strongest candidate that emerged from our computations was Signal Transducer and Activator of Transcription-6 (Stat6), a transcription factor regulated by IL-4 and IL-13. The roles of IL-4 and Stat6 in modulating lysosomal gene expression were evaluated in a primary cell culture model of alternatively activated mouse macrophages using data based on gene expression profiling, quantitative PCR and chromatin immunoprecipitations. Results obtained with macrophages from wild-type and Stat6-deficient mice demonstrate that Stat6 positively regulates a large number of lysosomal genes in an IL-4-dependent manner.

## Results

### Identification of transcriptional networks through correlation analysis

Previous studies have shown that the mRNA levels of transcriptional regulators are often predictive of the expression of their target genes [31-36]. Based on this premise, we asked whether mRNA correlation analyses across multiple datasets might reveal novel regulators of lysosomal gene expression.

Calculations were performed using expression profiles based on specific mouse and human Affymetrix microarray platforms for which large numbers of independent datasets are available at the NCBI GEO repository [37]. We then processed these files to generate average expression values for named, full-length mRNAs. A list of known transcription factors was assembled from gene ontology (GO) annotations and the literature [38,39].

**Figure 1:**
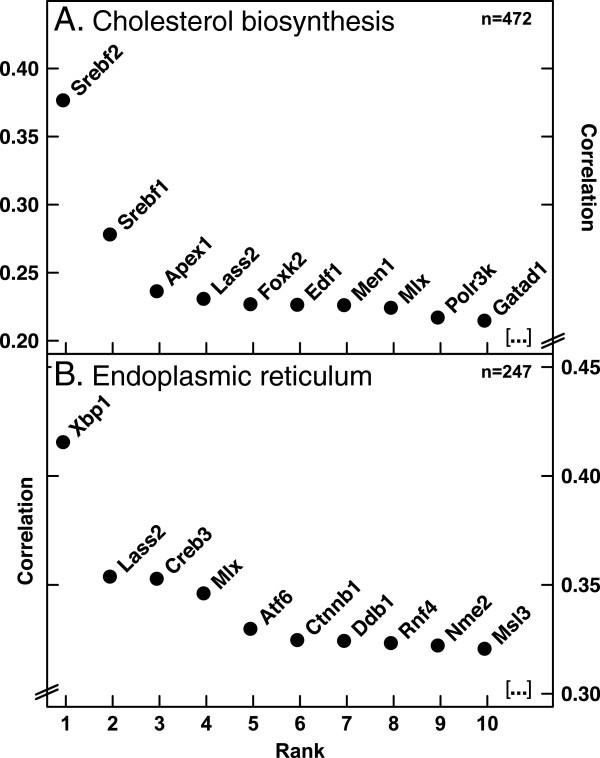
**Gene expression correlation analyses identify transcriptional regulators in the endomembrane system.** Analyses are based on 1,517 sets of publicly available mouse microarray expression profiles (Affymetrix Mouse_430_2 arrays) that were processed as described in Methods [37]. **(A)** Pearson correlations were calculated between 19 cholesterol biosynthesis reference genes and a list of 1,683 known transcription factors [6,38,39]. Average correlation coefficients were calculated for each transcription factor. **(B)** Average correlations across datasets were calculated among a list of 778 ER-specific genes (Additional file 2). Hierarchical clustering was performed and the resulting dendrogram split into 6 clusters (Additional file 3). The cluster with the largest average, consisting of 97 ER genes, was then used to calculate correlations with known transcription factors as in (a). **(A,B)** Prior to calculating correlations with transcription factors, datasets were filtered to exclude those with an average coefficient of variation of less than 30% for the respective reference set such that only the indicated number n of data series were included for final analyses. Each plot shows the results for the 10 highest-ranking transcription factors; only DNA-binding proteins (GO:0003677) are shown. Complete results are given in Additional file 1.

To verify the usefulness of the processed expression data for extracting transcriptional regulators, we initially interrogated the datasets for two pathways whose regulation is already well understood. We began by calculating a matrix of Pearson correlations between 19 mouse genes in the cholesterol biosynthesis pathway and 1,683 known transcription factors. The results were aggregated according to transcription factor and ranked. The resulting list was led by *Srebf2*, which encodes the transcription factor SREBP-2, known in fact to be a pivotal regulator of cholesterol metabolism and of the genes in the reference set (Figure 1A; Additional file 1) [6,40].

To further trial this strategy on a pathway for organelle biogenesis, we selected a group of 97 highly coordinated ER genes and found that this cluster correlates most strongly with Xbp1, an established master regulator of the ER stress response and of ER biogenesis (Figure 1B; Additional files 1, 2 and 3) [4].

In a complementary approach, *Srebf2* and *Xbp1* were each used as reference to calculate expression correlations with 16,771 mouse genes. Of the 10 genes that correlated most strongly with *Srebf2*, five are involved in cholesterol metabolism (p = 2.8E-09, hypergeometric test), and of the 10 genes most strongly correlated with *Xbp1* nine encode proteins associated with the ER (p = 3.2E-10) (Additional file 4). These values are similar to results returned by the Netview tool, which returns lists of ‘nearest neighbors’ based on co-occurrence in expression quantile groups [22]; according to Netview, the sets of 10 nearest neighbors for *Srebf2* and *Xbp1* each include five genes associated with cholesterol metabolism or the ER, respectively (data not shown). Taken together, the above results confirm that correlation analyses of co-regulated gene groups across our processed datasets can identify transcription factors that coordinate their expression.

### Correlation analysis of lysosomal gene expression

We next asked which transcription factors might correlate with the expression of lysosomal genes. Calculations were performed for 1066 mouse and 1412 human DNA-binding transcription factors for which high-quality data are available in the processed microarray datasets. In each case the 500 highest-ranking correlators were then analyzed for GO set enrichment using the Bioconductor GOstats package [41]. The resulting tables were searched for the terms ‘lysosome’ or ‘vacuole’, returning 49 transcription factors that scored positive at a significance cutoff of p ≤ 0.001 and according to both mouse and human datasets (Additional file 5; a searchable database of the complete results is available at http://genecan.sgul.ac.uk). The 49 transcriptional regulators include two factors, MITF and TFEB, that have previously been shown to direct the expression of lysosomal genes during differentiation and autophagy, respectively [26-29,42]. Further validating the data is the presence of 7 regulators that are known to physically associate with the endomembrane system (*ATF6, HCLS1, LASS2, CREB3, NFE2L1, NFE2L2*, and *TSG101*). A majority (65%), however, have been implicated during embryonic development or differentiation (*CEBPA, CEBPB, CEBPD, DDIT3, EGR2, EPAS1, FOS, HCLS1, HDAC5, HHEX, IKZF1, IRF1, IRF8, LMO2, LYL1, MAF, MAFB, MAFG, MITF, MNT, NFE2L2, NR1H2, NR1H3, PPARG, RELA, SPIB, STAT1, STAT3, STAT5A, STAT6, TFEB, TSG101*). Also prominent are transcription factors involved in interferon signaling (*CIITA, IRF1, IRF2, IRF5, IRF8, IRF9, NR1H2, NR1H3, PPARG, STAT1, STAT2*).

**Figure 2:**
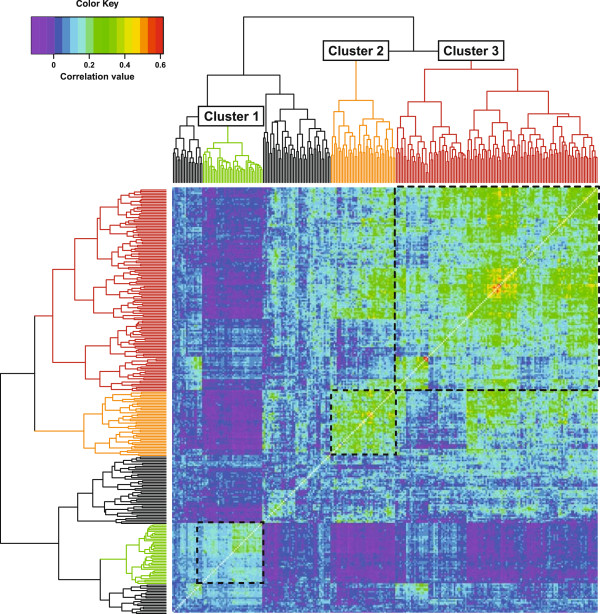
**Gene expression correlation analysis identifies distinct subsets of lysosomal genes.** Pearson correlation coefficients across 1,444 Mouse430_2-based microarray datasets were calculated among a list of 269 lysosomal genes that had been selected based on GO annotations (GO:0005764) and reviews of the lysosomal proteome [38,43,44]. The resulting data matrix (Additional file 6) was clustered, displayed as a heat map and the dendrogram split into branches as described in Methods. Clusters discussed in the main text are indicated.

The above results suggest that lysosomal gene sets are re-programmed in the context of different transcriptional networks. We therefore sought to identify subsets of lysosomal genes that are likely to be coordinately regulated. Correlations were calculated among a list of 269 lysosomal genes that had been assembled from GO annotations and reviews of the lysosomal proteome [38,43,44]. Computations were performed across 1,444 mouse datasets, and the genes were clustered hierarchically according to average correlation coefficients. Inspection of the resulting dendrogram and heat map identify three principal clusters, suggesting that the genes in each of these groups are often controlled collectively (Figure 2; Additional files 6 and 7).

Cluster 1 consists of 38 genes encoding lysosomal proteins whose functional profiles are generally similar to that of the entire lysosomal gene set. However, an unexpectedly large number of Cluster 1 genes (58%) have also been found at the plasma membrane (GO:0044459) or extracellularly (GO:0005576); of lysosomal genes outside of cluster 1, only 33% fall into either of these categories.

Cluster 2 consists of 41 genes that cover a range of different lysosomal functions, but subunits of the vacuolar H^+^ ATPase (*Atp6ap2, Atp6v1a, Atp6v1b2, Atp6v1c1, Atp6v1d, Atp6v1g1, Atp6v1h*) and components of the endo/lysosomal trafficking machinery (*Lamp2, M6pr, Rab14, Rab7, Rab9, Snap23, Vamp7, Vps4b*) are particularly prominent. The expression of genes in Cluster 1 is correlated negatively with 78% of Cluster 2, suggesting that these gene sets are frequently regulated reciprocally.

Cluster 3, the largest coherent group, includes 128 lysosomal genes whose protein products are involved in all aspects of lysosome physiology, including hydrolysis, acidification, transport and antigen presentation.

Collectively, the preceding analyses support the conclusion that distinct subsets of lysosomal genes can be coordinately regulated, hinting at the existence of dedicated transcriptional networks that control the expression of these clusters. The relatively low average correlation values indicate that such networks would be active only in a subset of physiological contexts.

### Transcription factors co-regulated with lysosomal syn-expression groups

**Figure 3:**
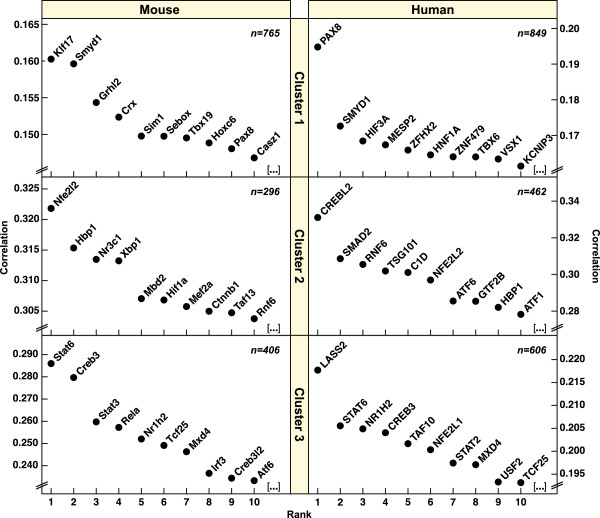
**Correlation of lysosomal clusters with transcription factors.** Lysosomal gene clusters identified in Figure 2 were used as reference sets for correlation analyses with known transcription factors as in Figure 1. Left panels, Mouse430_2 datasets. Right panels, human HG_U133_Plus2 datasets. Each plot shows the results for the 10 highest-ranking transcription factors; only DNA-binding proteins (GO:0003677) are shown. Complete results are given in Additional file 7.

Next, in order to explore the contexts and possible regulation of the three lysosomal gene clusters, each group was used as reference set for correlation analyses with known transcription factors across both mouse and human datasets. The complete results of these calculations are given in Additional file 7, and the ten highest-ranking transcription factors are listed in Figure 3.

A large proportion of the transcription factors that correlate with Cluster 1 are known to play roles in embryonic development and morphogenesis (*CRX, GRHL2, HNF1A, HOXC6, MESP2, PAX8, SIM1, SMYD1, TBX19, TBX6*; GO:0032502). Based on Pubmed literature searches, none have been associated with the regulation of lysosomal or vacuolar function. Two genes, *Pax8* and *Smyd1*, are common to both the mouse and human top-10 lists. Pax8 is important for thyroid and kidney development [45,46]. Twenty-nine percent of Cluster 1 genes (*Aldob, Aqp2, Atp6v0a4, Atp6v1b1, Atp6v1c2, Cckar, Cubn, Dnase1, Kcne1, Slc30a2, Sult1c2*) are highly expressed in mouse kidney (> 2 standard deviations above the mean) [47], which may help explain the moderate correlation of *Pax8* with the Cluster 1 gene set. *Smyd1* encodes a skeletal muscle and heart-specific histone methyltransferase, also known as Bop, that acts as a transcriptional repressor [48,49]. Whether Smyd1 contributes to the repression of the lysosomal Cluster 2 genes is currently not known.

Five of the top-10 transcription factors that correlate with Cluster 2 (*NFE2L2, HBP1, XBP1, HIF1A, CTNNB1*) have been linked to both oxidative stress and autophagy. The intersection of the mouse and human regulators contains three genes (*NFE2L2, HBP1 and RNF6*), with *NFE2L2* ranking highest. The *NFE2L2* gene encodes the protein nuclear factor-erythroid 2-related factor 2 (Nrf2), a basic-leucine zipper transcription factor that activates genes with antioxidant response elements (AREs) under conditions of oxidative stress [50]. Based on published chromatin immunoprecipitation-sequencing (ChIP-Seq) data from mouse embryonic fibroblasts (MEFs), Nrf2 binds to the promoters of five of the cluster 2 genes (*CD164, CLN5, CTSO, SCPEP1, TMEM55A*) (p = 0.014, hypergeometric test) and to eight additional lysosomal genes [51]. In lymphoid cells Nrf2 binds to the promoter of one of the cluster 2 genes (*IDS*) plus nine other lysosomal genes [52], with *FNBP1* and *GABARAPL1* being recognized in both MEFs and lymphoid cells. According to published microarray studies comparing wild-type and Nrf2-deficient mouse tissues, the expression of only a small and varying number of Cluster 2 genes are affected by Nrf2 in liver, small intestine and prostate [53-56]. Consistent across independent studies is the Nrf2-dependent regulation of *Ctsb* (cathepsin B) in liver, small intestine and prostate [54,56]; moreover, Nrf2 binds close to the *Scpep1* (serine carboxypeptidase 1) gene in MEFs and regulates its expression in liver and small intestine [51,54]. In summary, Nrf2 regulates a small number of lysosomal genes in a tissue-specific manner, but available evidence does not yet support the concept that Nrf2 accounts for the coordinate regulation of a majority Cluster 2 genes.

The transcription factor whose expression profiles correlated most strongly with those of the lysosomal Cluster 3 was mouse *Stat6*, and following an analogous analysis of human datasets, *STAT6* ranked near the top as well (Figure 3). Also correlating strongly with Cluster 3 are other regulators of immune function (Irf3, Nr1h2, Rela, Stat2, Stat3) and transcription factors bound to the membrane of the ER (Atf6, Creb3, Creb3l2, Lass2, Nfe2l1).

Given its strong correlation with the largest lysosomal cluster, Stat6 was chosen for further in-depth analyses.

### Stat6

**Figure 4:**
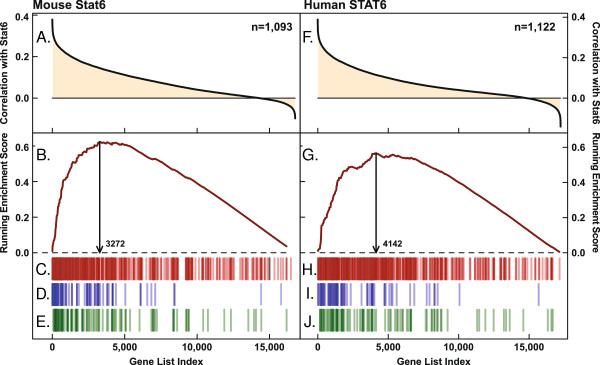
**Correlation of Stat6 with lysosomal genes.** Average expression correlations across the indicated number (n) of datasets were calculated between *Stat6* and each of **(A)** 16,771 genes represented on Affymetrix Mouse_430_2 arrays and **(F)** 17,236 genes represented on HG_U133_Plus2 arrays (see Methods). Only datasets with a coefficient of variation for *Stat6* expression of at least 5% were used for analyses. Genes were sorted according to correlation values, and the ranked lists were analyzed using the GSEA tool [57]. Gene set reference files in gene matrix transposed (gmt) format were generated by reformatting lists, downloaded from the AmiGO gene ontology website, containing all mouse and human GO associations (all gene product types and data sources) [38]. Complete results of GSEA and GOstats statistics are given in Additional file 8. **(A,F)** Average expression correlations with *Stat6*. **(B,G)** Running-sum enrichment scores for association with the gene set ‘lysosome’ (GO:0005764; 236 and 230 genes in mouse and human gene sets, respectively); arrows indicate the position of the maximum enrichment score and delimit the ‘leading-edge’ set of genes that account for the enrichment signal associated with the GO term ‘lysosome’ [57]. **(C,H)** Barcode plots indicate the positions of lysosomal genes along the rank lists. **(D,I)** Ranks of lysosomal genes that are positively regulated by Stat6 in IL-4 treated mouse macrophages (see Figure 5 and Additional file 9). **(E,J)** Ranks of lysosomal gene loci bound by Stat6 in mouse macrophages based on data deposited by Ostuni et al. (2013) [58] (see main text). Lines in barcode plots are drawn at 40% transparency.

As a cutoff-free approach to identifying which GO categories best correlate with *Stat6* we used the Gene Set Enrichment Analysis (GSEA) tool developed by Subramanian et al. [57]. GSEA calculates enrichment scores that reflect to what degree members of a particular GO category are concentrated at the extreme of a ranked gene list. The method also defines a ‘leading-edge’ subset of genes that account for the core of the enrichment score. The greatest enrichment scores for genes that correlate with mouse *Stat6* were seen for the GO terms ‘lysosomal membrane’ and ‘lysosome’ (nominal p = 0; Figure 4B and C; Additional file 8). In the human datasets the highest-scoring categories are related to type I interferon and Toll-like receptor signaling; however, the GO sets ‘lysosomal membrane’ and ‘lysosome’ also ranked high (nominal p = 0; Figure 4G and H; Additional file 8). The ‘leading-edge’ subsets for the GO term ‘lysosome’ contained 134 mouse and 126 human lysosomal genes, respectively (Figure 4C and H), of which 98 were common to both sets (73-78% overlap; Additional file 8). These results confirm that Stat6 mRNA levels are often coordinated with those of multiple lysosomal genes.

### IL-4 and Stat6 regulate lysosomal gene expression in macrophages

**Figure 5:**
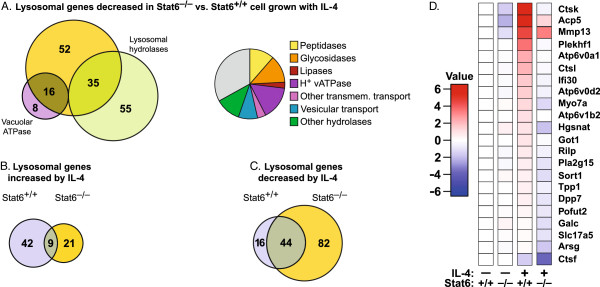
**Effects of IL-4 exposure and Stat6 deficiency on lysosomal gene expression in mouse macrophages.** Analyses are based on gene microarray data that have been previously described [64] [GEO:GSE25088]. RNA was from wild-type or *Stat6*-deficient mouse bone marrow macrophages cultured in triplicate for 10 days with 20 ng/ml M-CSF minus or plus 20 ng/ml IL-4. Gene expression profiles were obtained with Affymetrix Mouse_430_2 chips. Here, raw data were background-corrected, normalized and converted to log (base 2) fold changes as described in Methods. Detailed results are given in Additional file 9. **(A-C)** Venn diagrams were generated using Biovenn [65]. **(D)** Heat map indicating expression levels for lysosomal genes decreased at least two-fold in *Stat6*^-/-^ versus *Stat6*^+/+^ macrophages grown in the presence of IL-4. Log (base 2) changes in expression are given relative to those in wild-type cells grown without IL-4.

Stat6 is a member of the ‘signal transducer and activator of transcription’ family and expressed in most tissues [47,59]. The principal activators of Stat6 are IL-4 and IL-13, whose binding to cognate receptor complexes leads to recruitment of JAKs and JAK-mediated Stat6 phosphorylation, whereupon Stat6 dimerizes, moves to the nucleus and binds to specific promoter elements [60,61]. In addition to being stimulated by IL-4 and IL-13, Stat6 can become active in response to other cytokines and certain pathogens [60,62,63]. To obtain a detailed view of lysosomal gene regulation by IL-4 and Stat6, we analyzed IL-4-induced changes of gene expression in macrophages from wild-type and Stat6-deficient mice [64]. Detailed results are given in Additional file 9 and are graphically summarized in Figure 5. In the absence of IL-4 the Stat6 genotype had no effect on lysosomal gene expression, as would have been expected. However, in cells exposed to IL-4 an absence of Stat6 was associated with significant (p ≤ 0.05) changes in the expression of 149 (55%) known lysosomal genes. Comparing gene expression in Stat6-deficient versus wild-type cells, 46 lysosomal genes were increased and 103 lysosomal genes were decreased (Additional file 9).

The 103 lysosomal genes that are positively regulated by Stat6 encode proteins involved in all aspects of lysosomal function, including 39% of known lysosomal hydrolases, most subunits of the vacuolar H^+^ ATPase, components of the vesicular transport machinery and others (Figure 5A). Of these 103 genes, 53% are among the leading edge of mouse lysosomal genes whose expression correlates most significantly with Stat6 (Figure 4D; p = 0.031).

In wild-type macrophages exposed to IL-4, fifty-one lysosomal genes were induced and 60 lysosomal genes were suppressed (Figure 5B and C), reflecting a complex reconfiguration of gene expression in this cell type [58,64]. The contribution of Stat6 to lysosomal gene expression, however, is generally positive: in Stat6-deficient cells the activating effects of IL-4 were almost completely abolished (Figure 5B), whereas the suppressing effects of IL-4 largely persisted (Figure 5C). Surprisingly, 82 lysosomal genes were suppressed by IL-4 in Stat6-deficient, but not in wild-type cells (Figure 5C), indicating that Stat6 usually antagonizes the inhibitory effect of IL-4 on the expression of these genes.

**Figure 6:**
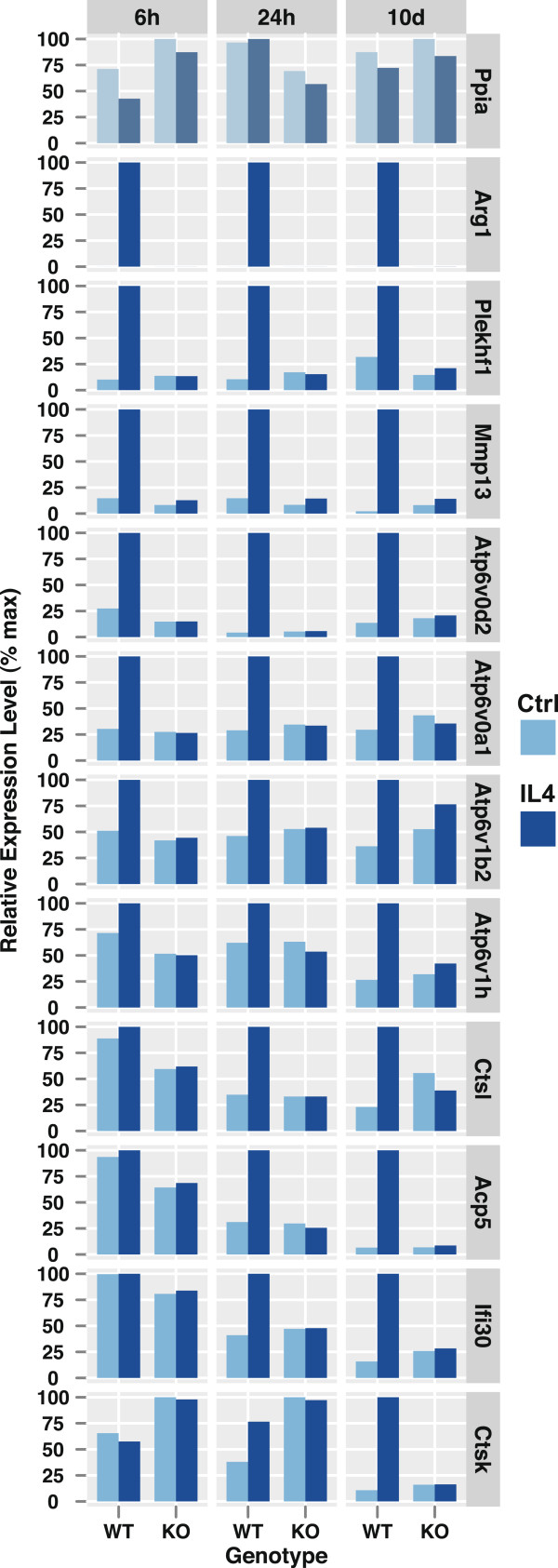
**Effects of IL-4 and Stat6 on lysosomal gene expression as measured by quantitative PCR.** RNA was obtained from triplicate cultures of wild-type or *Stat6*-deficient mouse bone marrow macrophages. For the panels in columns 1 and 2 macrophages were cultured for 5 days with 20 ng/ml M-CSF and then with 20 ng/ml IL-4 for an additional 6 or 24 hours as indicated. The data in column 3 were obtained from cultures grown ± M-CSF and IL-4 for 10 days as in Figure 5. RNA was reverse transcribed and analyzed by quantitative PCR as described in Methods. Oligonucleotides are listed in Additional file 13.

Next we sought to verify the microarray data through an alternative method. For this, we cultured bone marrow macrophages from wild-type and Stat6-deficient mice with IL-4 for 6 hours, 24 hours or 10 days, and RNA was extracted for reverse transcription and quantitative PCR. Most targets were chosen from among the lysosomal genes that, according to Figure 5D, were decreased at least two-fold in *Stat6*^-/-^ versus *Stat6*^+/+^ macrophages grown in the presence of IL-4. Expression of the *Ppia* gene (encoding cyclophilin A) remained relatively unchanged under the four conditions and was used to normalize values for the remaining genes. The *Arg1* gene (encoding arginase I), an established target of IL-4/Stat6 signaling [66,67], was strongly induced by IL-4 in wild-type cells, but its expression remained low in Stat6-deficient cells (Figure 6). Similar results were obtained for most of the lysosomal genes that were analyzed, with the strongest regulation by IL-4 seen for *Mmp13, Acp5, Ctsk, Atp6v0d2, Ifi30* and *Ctsl*. Only *Myo7a* and *Hgsnat*, which appeared moderately induced by IL-4 based on microarray analyses (Figure 5D), changed much less significantly according to the qPCR data (data not shown). Overall, however, the qPCR results are in good agreement with the microarray data and confirm that IL-4 controls the expression of multiple lysosomal genes in a Stat6-dependent manner.

### Stat6 binds close to lysosomal gene loci

To explore whether Stat6 might bind to genomic loci encoding lysosomal genes, we interrogated publicly available ChIP-seq data from IL-4 treated macrophages [58]. According to peak coordinates from macrophages grown with IL-4 for 4 hours, Stat6 binds in the vicinity of 4,520 named genes (± 5 kb of transcription start site), 153 of which have been annotated as encoding lysosomal proteins (p = 6E-62, hypergeometric test).

As an unbiased approach to determining whether genes associated with specific functions are statistically overrepresented among the 4,520 Stat6-bound targets, the list was subjected to gene set enrichment analysis using GOstats [41]. Excluding categories with more than 1000 members, the three highest-ranking categories returned by this analysis were ‘ribosomal subunit’ (GO:0044391; p < 6E-11), ‘cytosolic part’ (GO:0044445; p < E-10) and ‘lysosome’ (GO:0005764; p < 2E-10) (Additional file 10). These data indicate that genes encoding lysosomal proteins make up a significant fraction of the genomic loci that Stat6 physically interacts with.

Of the 153 lysosomal genes whose loci are bound by Stat6 according to the current measure, 46% are among the genes whose mRNA levels were significantly reduced in Stat6-deficient macrophages grown with IL-4 (see Figure 5 and Additional file 9). However, this fraction increases to 72% if Stat6 peaks located anywhere inside a gene are also counted as targets.

**Figure 7:**
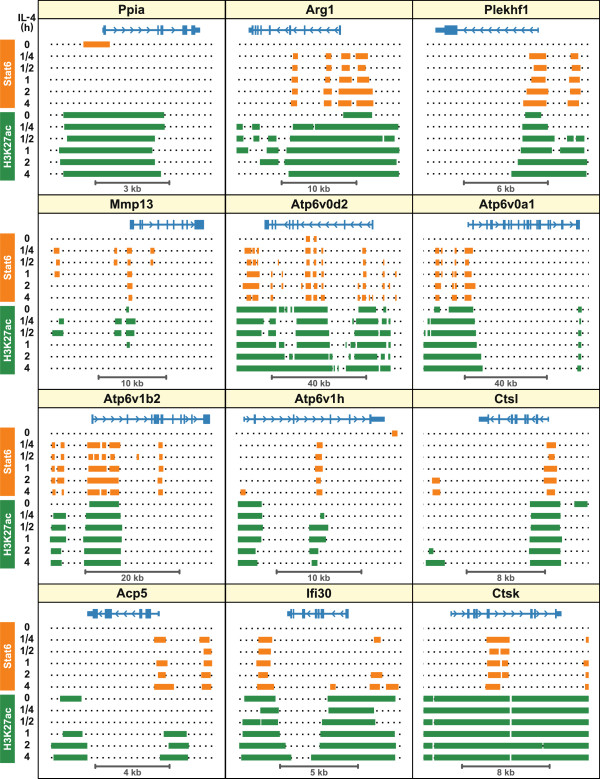
**Stat6 binds to lysosomal gene loci in mouse macrophages.** Analyses are based on publicly available ChIP-seq data deposited by Ostuni et al. (2013) [GEO:GSM1022294, GSM1022295, GSM1022296, GSM1022297, GSM1022298, GSM1022299, GSM1022300, GSM1022301, GSM1022302, GSM1022303, GSM1022304, GSM1022305] [58]. In that study mouse bone marrow-derived macrophages were grown with IL-4 for the indicated time, chromatin was immunoprecipitated with antibodies against Stat6 or H3K27ac, and the DNA fragments were sequenced. Here, data files with peak coordinates were annotated and aligned to selected genomic loci as described in Methods. Coding regions of the indicated genes are shown in blue, with the direction of transcription indicated by arrow heads. In some cases, neighboring genes or transcript variants were omitted for clarity. Peak regions for Stat6 and H3K27ac are shown in orange and green, respectively.

In order to study the kinetics of Stat6 binding to lysosomal loci, Stat6 ChIP-seq peaks from different time points after IL-4 addition were aligned to genomic maps of the genes whose mRNA levels were analyzed in Figure 6. As expected, no IL-4 induced Stat6 binding could be seen around the *Ppia* locus, whereas several Stat6 peaks appeared at the *Arg1* gene as early as 15 min after IL-4 addition (Figure 7). Similarly, IL-4 rapidly induced the binding of Stat6 to all of the 10 lysosomal genes that are shown, and binding patterns remained relatively stable for the subsequent 4 hours. At the *Atp6v1h*, *Ctsl* and *Ifi30* loci additional Stat6 peaks appear at later time points, suggesting contributions of co-factors whose activities might increase with delayed kinetics. To independently verify the Stat6 ChIP-seq data, several peaks were selected for verification by ChIP-qPCR, and in each case the results were confirmatory (Additional file 11). Collectively, mRNA quantifications and ChIP-seq data show that Stat6-mediated activation of lysosomal gene expression coincides with the binding of Stat6 to the affected promoters, strongly suggesting that Stat6 plays a direct role in augmenting the transcription of these targets.

### Stat6 sites at lysosomal genes are near active chromatin

**Figure 8:**
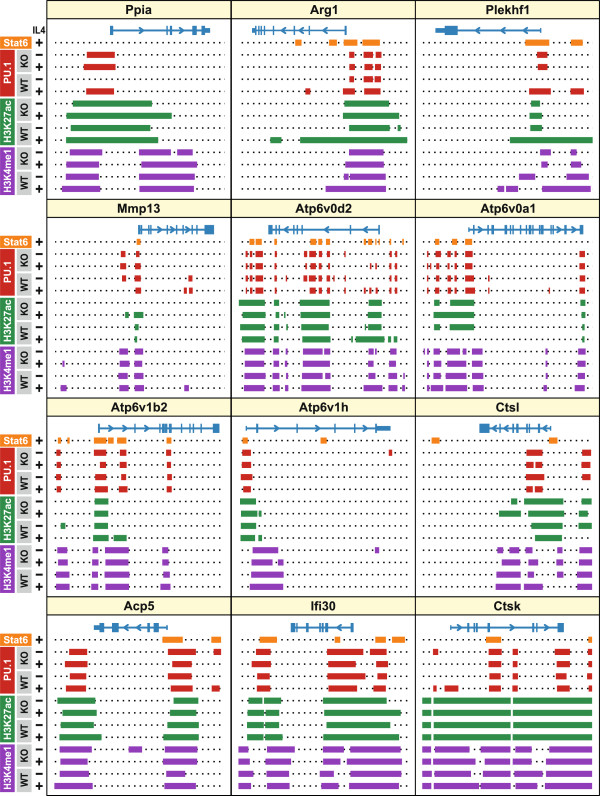
**Effects of IL-4 and Stat6 on chromatin modifications near lysosomal genes.** Analyses are based on publicly available ChIP-seq data deposited by Ostuni et al. (2013) [GEO:GSM1022256, GSM1022257, GSM1022258, GSM1022259, GSM1022264, GSM1022265, GSM1022266, GSM1022267] [58]. In that study bone marrow-derived macrophages from wild-type and Stat6-deficient mice were grown ± IL-4 for 4 hours, chromatin was immunoprecipitated with antibodies against Pu.1, H3K27ac and H3K4me1, and the DNA fragments were sequenced. Here, peak coordinates were annotated and aligned to selected genomic loci as in Figure 7. Peak regions are shown in orange (Stat6), red (Pu.1), green (H3K27ac) and purple (H3K4me1). Genomic regions and scales correspond to those shown in Figure 7.

Actively transcribed genes are often controlled through promoter and enhancer elements characterized by binding sites for multiple, tissue-specific transcription factors and by the presence of nucleosomes with activating epigenetic modifications such as monomethylated histone H3 lysine-4 (H3K4me1) and acetylated H3 lysine-27 (H3K27ac) [68,69]. In macrophages, cell type-specific gene expression depends in part on Pu.1, an ETS-domain transcription factor required for the development of myeloid and B-lymphoid cell types [70-72]. Pu.1 has previously been shown to cooperate with Stat6 in controlling the expression of several genes [61]. To characterize Stat6-bound genomic regions near lysosomal genes, we studied publicly available ChIP-seq data to explore the co-localization of Stat6 with H3K4me1, H3K27ac and Pu.1 in samples from wild-type and Stat6-deficient macrophages that had been grown ± IL-4 for 4 hours [58]. As a point of reference we selected 196 Stat6 peaks that were observed consistently after 1, 2 and 4 hours of IL-4 exposure and within 5 kb of the transcription start sites of lysosomal genes (133 lysosomal genes; range, 1–4 peaks per gene; average, 1.5 peaks per gene) (Additional file 12). Of these 196 peaks, 75% were marked by the presence of H3K4me1, H3K27ac and Pu.1 in wild-type cells grown without IL-4, showing that Stat6 predominantly binds to regions characterized by activating epigenetic markers (Figure 8 and Additional file 12).

In macrophages, latent enhancers have been defined as sites devoid of H3K4me1, H3K27ac and Pu.1 that acquire H3K4me1 upon stimulation [58], and none of the Stat6 peaks near lysosomal genes fall into this category (Additional file 12).

Enhancer elements containing H3K4me1, but no H3K27ac, have been described as “poised” for activation [73,74]. Among the regions bound by Stat6 near lysosomal genes, 86% (169/196) have pre-existing nucleosomes containing H3K4me1, but only 4% (8/196) contain H3K4me1 alone and acquire H3K27ac upon IL-4 exposure; at 5 of these sites (near *Atp6v0d2, Htt, Ids, Plekhf1, Srgn*) the IL-4 induced acetylation of H3K27 was dependent on Stat6 (Figure 8 and Additional file 12).

H3K27ac marks are usually concentrated near promoters and their presence is predictive of gene expression [75]. Inspection of the data in Additional file 12 shows that 89% (174/196) of the lysosomal Stat6 peak regions already contain H3K27ac in untreated wild-type cells. We identified 9 lysosomal Stat6 peak regions at which H3K27ac was induced by IL-4, and this modification was Stat6-dependent near the same 5 genes at which IL-4/Stat6 promote monomethylation of H3K4 (*Atp6v0d2, Htt, Ids, Plekhf1, Srgn*), indicating that Stat6 coordinates activating chromatin modifications at these promoters. Two of the affected targets, *Atp6v0d2* and *Plekhf1*, are among the lysosomal genes whose mRNA levels are most strongly regulated by IL-4 and Stat6 (Figures 5, 6, 7 and 8). At many of the lysosomal genes whose expression is controlled by Stat6, IL-4 exposure led to a pronounced expansion of pre-existing H3K27ac marks around the Stat6 peaks (e.g. *Arg1, Plekhf1, Atp6v0a1, Atp6v1b2, Atp6v1h, Ctsl, Acp5*), and at most of these sites the spreading of H3K27ac was dependent on Stat6 (Figures 7 and 8). In summary, Stat6 binds near lysosomal genes at sites marked by active chromatin configurations, and at several lysosomal genes Stat6 contributes to the establishment or expansion of these markers. These results further strengthen the concept that Stat6 plays pivotal roles in activating the expression of lysosomal genes in macrophages.

## Discussion

In the current study we used gene expression correlation analyses to search for DNA-binding transcription factors whose activities might relate to lysosomal function. The strongest candidate that emerged from our data was Stat6, a widely expressed transcription factor that is activated in response to specific cytokines and pathogens. In support of a role for Stat6 upstream of lysosomal gene expression we demonstrate that IL-4 induced Stat6 positively regulates a wide range of lysosomal genes in mouse macrophages.

Our *in silico* strategy was based on a large body of work showing that the expression of transcription factors and their target genes are often positively related [31-36]. If the expression of a group of lysosomal genes was transcriptionally coordinated through the action of a transcription factor, we reasoned, it might be possible to identify such a regulator through correlation analyses across a great number of microarray data. Association of transcriptional regulators with their target genes, based on expression data, has previously been demonstrated using a number of methods, including mutual information scoring [33,76], probabilistic methods [34], differential equations [77], Gibbs sampling [78] and Spearman correlations [31]. Here, we applied a simplified clustering approach by calculating Pearson correlations between lists of known transcription factors and potential target genes. Correlation values were averaged across hundreds of expression datasets, and genes were ranked accordingly. In one round of calculations genes most highly correlated with individual known transcription factors were screened for GO set enrichment which, at a cutoff of p ≤ 0.001, produced a list of 49 DNA-binding regulators whose expression is highly correlated with lysosomal genes. In a complementary approach transcription factors were sorted according to their correlation with groups of co-regulated lysosomal genes.

The list of 49 regulators includes MITF and TFEB, two proteins that have been previously identified as regulators of lysosomal gene expression in the context of autophagy, and differentiation [26-29,42]. Further validating our approach was the presence of proteins that are known to be physically associated with compartments of the endomembrane system, including ATF6, HCLS1, LASS2, CREB3, NFE2L1, NFE2L2, and TSG101. Strikingly, 65% of the 49 regulators have been implicated during embryonic development or differentiation, and 22% are involved in interferon signaling, suggesting that these processes are frequently accompanied by reconfiguration of the lysosomal system. Augmented expression of lysosomal genes during development and differentiation might support the generation of tissue-specific, lysosome-related organelles [79]. Moreover, endo/lysosomal factors are increasingly being implicated during cell migration and polarity, both important aspects of development, as well as of wound healing and cancer [80]. Positive correlation between lysosomal and interferon signaling genes points to the front-line role of lysosomes in the defense against pathogens [81-83].

Cluster analysis of all known lysosomal genes led to the identification of several subgroups whose expression appears to be coordinated. Unexpectedly, one cluster was characterized by being correlated negatively with a large fraction of other lysosomal genes, indicating that the expression of these groups is often mutually exclusive. The mechanistic origin and physiological relevance of these results is not yet understood. The largest lysosomal cluster includes large fractions of known acidic hydrolases and vacuolar H^+^ ATPase subunits, supporting the impression from previous studies that core lysosomal functions are transcriptionally coordinated [21-23]. The transcription factor most strongly correlated with this cluster was Stat6. A member of the signal transducer and activator of transcription family [84], Stat6 is activated predominantly through JAK-mediated tyrosine phosphorylation in response to IL-4 or IL-13. Stat6-deficient mice are viable, but suffer defects in the differentiation of several immune and non-immune cell types, exhibit increased susceptibility to infection by certain viral, bacterial and helminthic pathogens and show attenuated allergic responses [61]. Conversely, ectopically activated Stat6 is frequently found in tumor samples [85-87].

In monocytes, Stat6 signaling promotes the differentiation into a class of alternatively activated macrophages [88]. Based on microarray data obtained from primary mouse macrophages cultured with IL-4, we found that the expression of 103 lysosomal genes was dependent on Stat6, reflecting 40% of the known lysosomal proteome and 54% of lysosomal genes expressed in this cell type; 45 of these genes have been associated with human diseases [89]. Of particular interest is the role of Stat6 in controlling the vacuolar H^+^ ATPase that, by virtue of maintaining the acidic pH in the endo/lysosomal system, is pivotal to all aspects of lysosomal function [90]. Of 15 different subunits and associated factors that make up the vacuolar H^+^ ATPase [90,91], 14 were found to be controlled by Stat6, and three subunits (encoded by *Atp6v0a1*, *Atp6v0d2* and *Atp6v1b2*) were among the lysosomal genes most strongly induced by IL-4.

Previous studies have shown that IL-4 increases the expression of the lysosomal proteases cathepsin L and S in mouse macrophages and of cathepsin S in human bronchial and conjunctival epithelial cells [92-94]; however, the transcription factor responsible for this regulation had not yet been identified. Through analysis of microarray data we found that in mouse macrophages exposure to IL-4 augments the expression of eight lysosomal protease genes, and in 7 of these cases (*Ctsk, Ctsl, Ctso, Ctsz, Mmp13, Scpep1, Tpp1*) the IL-4 effect was dependent on Stat6. In total, the IL-4/Stat6 system was found to control the expression of 39% of known lysosomal hydrolases, a group that in addition to proteases, include glycosidases, lipases and other degradative enzymes. Genes involved in vesicular targeting to lysosomes and in the movement of substances across the lysosomal membrane are also regulated by IL-4/Stat6. These effects are likely to contribute to the heightened influx of endocytic substrates and the increased capacity for lysosomal degradation that have previously been observed in IL-4 treated macrophages [94-96].

Alternatively activated macrophages have been implicated in tissue repair [97], and we speculate that IL-4/Stat6-mediated expression of lysosomal enzymes may facilitate the repair-associated turnover of extracellular matrix, for example through secretion of acidic hydrolases into the extracellular space [20,98], or through intracellular digestion of phagocytosed collagen fibrils [99]. In support of this model, the lysosomal genes that are most strongly affected by IL-4 and Stat6 in macrophages, encoding cathepsins L and K, tartrate-resistant acid phosphatase, collagenase-3 and vacuolar H^+^ ATPase, have all been shown to play important roles in extracellular matrix degradation [98,100-102]. Furthermore, Stat6-controlled expression of several lysosomal and extracellular proteases has been implicated in tissue destruction during pulmonary emphysema and is thought to contribute to the invasiveness of glioma tumours [103-105].

In wild-type macrophages IL-4 effects a complex reprogramming of gene expression, with similar numbers of lysosomal genes being induced and suppressed. Stat6, however, appears to mediate only the activating effects of IL-4 on lysosomal mRNAs. In macrophages devoid of Stat6 the induction of lysosomal genes by IL-4 was blocked or severely reduced. On the other hand, in *Stat6*^-/-^ cells the suppressive effects of IL-4 remained largely unchanged, pointing to an IL-4 induced signaling branch that operates independently of Stat6. Similar interdigitation of IL-4 and Stat6 signaling has been observed in the context of Th2 differentiation [106,107].

Unexpectedly, the expression of 82 lysosomal genes was reduced by IL-4 in Stat6-deficient, but not in wild-type cells. Stat6 thus acts both to mediate increased expression as well as to counteract an unknown, inhibitory pathway that is triggered by IL-4. These results reveal that the role of Stat6 in this context is significantly broader than might have been expected from the number of IL-4 induced genes alone. The mechanism behind this effect is not yet known. Binding of Stat6 to affected promoters might for example block access or regulation by IL-4-induced inhibitory factors, compensate for the loss of other positive regulators, or counter the effects of repressive epigenetic alterations. Stat6 deficiency in mouse T cells has been shown to cause a marked increase of trimethylated lysine-27 on histone 3 (H3K27me3), indicating that Stat6 supports transcription in part by antagonizing inhibitory chromatin modifications [106]. However, to what degree Stat6 exerts this effect in macrophages still has to be explored in detail.

ChIP data show that about 70% of the lysosomal loci that are regulated by Stat6 in macrophages have nearby Stat6 binding sites, indicating that Stat6 is likely exerting proximal effects in directing the expression of these targets. A similar number of Stat6 sites near lysosomal genes have been identified in a ChIP-seq analysis in mouse Th2 cells; however, very few of these genes were induced on the mRNA level [106]. While phosphorylation on tyrosine 641 is sufficient to allow the binding of Stat6 dimers to cognate DNA elements, Stat6 is unable to activate transcription on its own [106-108], and it must cooperate with other proteins to promote gene expression [61]. In this context, activating chromatin modifications are likely to be important, as most Stat6 peaks overlap with regions of histone H3 acetylation, and the most strongly regulated Stat6 targets experience extensive expansions of H3K27ac marks in an Il-4 and Stat6-dependent manner. Exactly what additional factors are involved in IL-4/Stat6-controlled lysosomal gene expression, and whether they act in concert with or downstream of Stat6 remains to be studied.

## Conclusions

**Figure 9:**
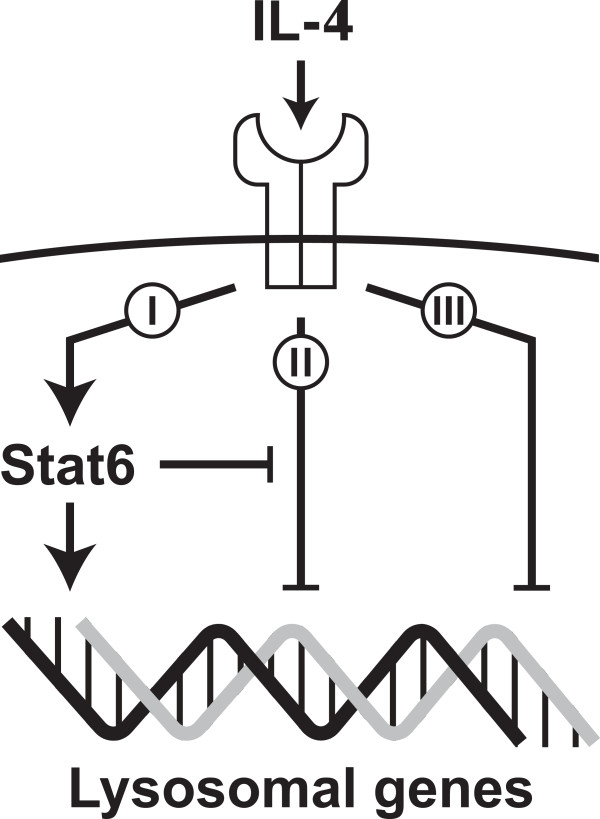
**Principal mechanisms of lysosomal gene regulation by IL-4 and Stat6.** Analyses of gene expression data from wild-type and Stat6-deficient mouse macrophages (Additional file 9 and Figure 5) support a model in which lysosomal genes are regulated through three main mechanisms. The expression of lysosomal genes in category I (n = 42) is induced by IL-4 in a pathway that depends on Stat6. Category II genes (n = 82) are suppressed by IL-4 in Stat6-deficient cells, but are induced or remain unchanged in wild-type cells, which points to IL-4 triggering at least two opposing regulatory signals. Lysosomal genes in catergory III (n = 44) are suppressed by IL-4 in a Stat6-independent manner. The mechanisms by which IL-4 suppresses lysosomal gene expression in this system are currently unknown.

Understanding how the structure and function of organelles are molded during embryonic development and differentiation is a major goal of cell and developmental biology. The aim of this study was to identify transcriptional networks that are associated with the re-programming specifically of lysosome-related genes. Through large-scale analyses of published microarray data we identified more than 50 DNA-binding transcription factors whose expression correlates with significant numbers of lysosomal genes. Affiliations identified in this manner indicate that mRNAs for lysosomal genes are frequently modulated in concert with regulators that are active during of differentiation, development, interferon signaling and oxidative stress, suggesting broad re-programming of lysosomal genes in these contexts. Depending on network structure, expression of transcription factors can correlate with their downstream target genes, and for most of the regulators identified here such directing roles in lysosomal gene control remains to be explored. However, Stat6, the strongest candidate emerging from our correlation analysis, was clearly identified as an upstream regulator for a large number of lysosomal genes in IL-4 treated mouse macrophages. According to the effects of IL-4, lysosomal genes can be grouped into three principal categories (Figure 9); lysosomal genes in category I are induced by IL-4 through a Stat6-dependent mechanism; genes in category II tend to be suppressed by IL-4, but this effect is negated in the presence of Stat6; category III genes are suppressed by IL-4 through a pathway operating independently of Stat6. In summary, this work illuminates the principal contexts of lysosomal gene regulation, identifies a novel pathway of lysosomal gene control and advances understanding of the cell and molecular biology of alternative macrophage differentiation.

## Methods

### Materials

Cell culture media were from PAA Laboratories (Yeovil, UK) and fetal bovine serum (FBS) was from Sigma Aldrich (Gillingham, UK).

### Expression correlation analyses

All data manipulations and calculations were performed with custom Unix/Linux or R version 2..0 scripts [109]. 1,517 microarray data series based on Affymetrix mouse genome 430 2.0 arrays (Mouse_430_2; Geo platform accession number GPL1261) and 1,744 data series based on Affymetrix Human Genome U133 Plus 2.0 arrays (HG_U133_Plus2; GEO platform accession number GPL570), each containing at least 6 samples, were downloaded from the NCBI GEO ftp site [37]. The data in each file were reduced to high-quality probe sets attributable to single, full-length mRNAs (labeled “grade A” in Affymetrix annotation files; Mouse430_2.na32.annot.csv and HG_U133_Plus_2.na32.annot.csv). As the number of probe sets on these platforms varies for individual targets, values for genes represented by more than one probe set were averaged, leaving data for 16,772 mouse and 17,237 human genetic endpoints. Each dataset was then saved as an R data frame in binary format for subsequent analyses. Gene expression correlation matrices were calculated using the R ‘cor’ function (Pearson method).

### Clustering

Data matrices were subjected to hierarchical clustering using the R ‘hclust’ function (complete method), and heat maps were generated with the ‘heatmap.2’ function from the gplots library [110]. Dendrograms were split into clusters using the R ‘cutree’ function.

### Microarray data analysis

For the analyses that resulted in Additional file 9 and Figure 5, raw data (cel files) were background-corrected and normalized using the Bioconductor GCRMA package [111]. Only probe sets based on full-length cDNAs, assigned to a single gene and annotated as “grade A” according to Affymetrix annotation files were used for further analyses. Probe IDs were replaced with gene symbols, and data for genes represented by more than one probe set were averaged. Genes with average expression values of less than one standard deviation (SD) under all four conditions were considered not expressed and excluded. In the remaining matrix (10,232 genes) values below 1 SD were set to 1 SD. Variances between pairs of triplicate data were then compared with an F test (R ‘var.test’ function); if the resulting p value was greater than 0.05, an unpaired Student’s t test was performed, otherwise Welch’s t test was used (R ‘t.test’ function). The p values returned by the t tests were corrected for multiple hypothesis testing using the false discovery rate method (R ‘p.adjust’ function). Log (base 2) fold changes were set to zero when p values returned by p.adjust were greater than 0.05.

### Gene sets

GO annotations are based on lists that we downloaded from the AmiGO gene ontology site [38]. Lists of known transcription regulators were based on a compilation published by Ravasi et al. [39] that was extended based on recent GO annotations (GO:0000981, GO:0000982, GO:0000983, GO:0000988, GO:0000989, GO:0001010, GO:0001011, GO:0001071, GO:0001076, GO:0001087, GO:0001133, GO:0003700, GO:0003705, GO:0004879). Lists of lysosomal genes were based on combined mouse and human annotations for the GO terms ‘lysosome’ (GO:0005764) and ‘vacuole’ (GO:0005773), which were extended based on reviews of the lysosomal proteome [38,43,44]. In some cases gene lists were further modified by removing genes for which no high-quality data were available in the given data set.

### Analysis of published ChIP-seq data

Files with peak coordinates in bed format were downloaded from the NCBI GEO depository [GEO:GSE38379]. The data were annotated using the Bioconductor ChIPpeakAnno package (v*ersion 2.6.1.*) [112], and peaks were graphically aligned to genomic loci using R code based on the Gviz (version 1.2.1.), GenomicFeatures (version *1.10.2.*) and Lattice packages [113-115].

### Promoter sequence analysis

Promoter sequences ± 2 kb relative to transcription start sites were searched for perfect matches to the Stat6 consensus binding motif (TTCNNNNGAA) using the RSAT tool (DNA-pattern method, 0 substitutions) [116,117].

### Mouse husbandry

Stat6-deficient mice (C.129S2-Stat6^tm1Gru/J^) were obtained from Jackson Laboratories [118]. Animals were housed in Tecniplast blue line IVC cages at a negative pressure of 25pa with 75 air changes an hour and fed a standard CRM diet (SDS Diets Services, Essex, UK). Work involving mice was approved by the UK Home Office and the University of Debrecen Medical and Health Science Center Ethics Committee (license number 120/2009/DE MAB), respectively.

### Cell culture

For studies performed in London, cells were cultured at 37°C in an atmosphere of 5% CO_2_. Medium A refers to RPMI supplemented with 10% FBS, 50 U/ml penicillin and 50 μg/ml streptomycin. To obtain bone marrow macrophages, the femurs of mice were submersed in PBS, crushed with hemostats, filtered through 70-μm cell strainers (BD Falcon), washed with PBS and plated in medium A on untreated 10-cm Petri dishes overnight. Unattached cells were then set up in 6-well plates at 5.5x10^6^ cells per well in medium B plus 20 ng/ml murine recombinant M-CSF (Peprotech) plus or minus murine recombinant IL-4 (Peprotech).

For work performed in Debrecen, cells were isolated and differentiated as previously described [119]. Bone-marrow was flushed from the femur of wild-type C57BI6/J male animals. Cells were purified through a Ficoll-Paque gradient (Amersham Biosciences, Arlington Heights, IL) and cultured in DMEM containing 20% endotoxin-reduced fetal bovine serum and 30% L929 conditioned medium for 5 days.

### RNA analysis by qPCR

Total RNA was isolated using Trizol reagent (Invitrogen) and 1–2 μg used as template in 20-μl reverse transcription reactions using Tetro Reverse Transcriptase (Bioline) or a Superscript III CellDirect cDNA synthesis kit (Invitrogen). Quantitative PCR reactions were performed in a Bio-Rad CFX96 thermocycler and set up using a Platinum SYBR Green qPCR Supermix (Invitrogen) or SYBR green dye from Diagenode, each in a total volume of 10 μl containing 0.5 μl cDNA and 200 nM primers. Primer sequences are given in Additional file 13. Standard curves with serial template dilutions were included with each run.

### Chromatin immunoprecipitation (ChIP)

ChIP was performed as previously described [120] with minor modifications. Briefly, cells were crosslinked with DSG (Sigma) for 30 minutes and then with formaldehyde (Sigma) for 10 minutes. After fixation chromatin was sonicated with a Diagenode Bioraptor to generate 200-1000 bp fragments. Chromatin was immunoprecipitated with pre-immune IgG (Millipore, 12–370), or with a polyclonal antibody against STAT6 (Santa Cruz, sc-981). Chromatin antibody complexes were precipitated with anti-IgA paramagnetic beads (Life Technologies). After 6 washing steps complexes were eluted and the crosslinks reversed. DNA fragments were column purified (Qiagen, MinElute). DNA was quantified with a Qubit fluorometer (Invitrogen). Immunoprecipitated DNA was quantified by qPCR and normalized to values obtained after amplification of unprecipitated (input) DNA.

### Graphics

Graphics were generated with custom R scripts, in some cases using extensions provided by the gplots, ggplot2 and other packages as indicated [110,121]. R-generated graphic files in portable document format (PDF) were further edited in Adobe Illustrator.

### Availability of supporting data

A searchable database with the results of transcription factor gene expression correlation analyses is available at http://genecan.sgul.ac.uk.

## Abbreviations

ChIP-seq: Chromatin immunoprecipitation-sequencing; ER: Endoplasmic reticulum; GO: Gene ontology; GSEA: Gene set enrichment analysis; H3K27ac: Acetylated histone H3-lysine-27; H3K4me1: monomethylated histone H3 lysine-4; HG_U133_Plus2: Affymetrix Human Genome U133 Plus 2.0 array; IL-4: Interleukin-4; IL-13: Interleukin-13; Mouse_430_2: Affymetrix mouse genome 430 2.0 array; qPCR: quantitative polymerase chain reaction; Stat6: Signal transducer and activator of transcription 6

## Competing interest

The authors declare that they have no competing interests.

## Authors’ contributions

AN conceived of the study, wrote computer programs and wrote the first draft of the manuscript. AN, LM, BD, ZC and NB performed tissue harvest. AN, BD, HS, IV, LB, LM and ZC cultured cells. AN, BD, IV, LB, LM, and ZC extracted RNA and performed quantitative PCR. BD, LN, and ZC performed chromatin immunoprecipitation. NB and SJ designed knockout mouse studies and managed animal husbandry. AN, LB and LM performed data analysis. LB, LM, LN, NB, and SJ helped write the final version of the paper. All authors read and approved the final manuscript.

## Authors’ information

Laszlo Nagy and Axel Nohturfft share senior authorship.

## Supplementary Material

Additional file 1 **Expression correlation between membrane biogenesis genes and known transcription factors.** The data pertain to Figure 1 of the main article, and calculations were performed as detailed in the accompanying legend. (Worksheet 1, “Cholesterol biosynthesis”) *Column A* lists 19 genes encoding enzymes in the cholesterol biosynthesis pathway that were used as a reference set [6]. *Column C* lists NCBI GEO accession numbers of 472 Mouse430_2-based gene expression data series that had been selected based on a minimum average coefficient of correlation among the 19 reference genes of 30%. The genes in *Column E* are based on a list of known transcription factors (see Methods). Values in *Column G* represent average Pearson correlation coefficients calculated between the corresponding transcription factor in *Column E* and the reference genes from *Column A* across the data series listed in *Column C. Column F* indicates whether the gene encodes a DNA-binding protein according to GO annotations (GO:0003677). Transcription factor genes are listed according to correlation values in descending order. (Worksheet 2, “ER”) Column A lists 95 highly correlated ER genes that have been selected as detailed in the legends to Figure 1b and Additional file 3, after removing known transcription factors. Column C lists NCBI GEO accession numbers of 247 Mouse430_2-based gene expression data series that had been selected based on a minimum average coefficient of correlation among the 95 reference genes of 30%. Columns E-G contain correlation data for transcription factor genes as detailed above. Click here for file

Additional file 2 **Matrix of expression correlations among ER genes in mouse.** This supplement pertains to Figure 1b and Additional file 3. Data were generated as described in the accompanying legends. Tab-delimited. Click here for file

Additional file 3 **Gene expression correlation analysis identifies distinct subsets of ER genes.** These data pertain to Figure 1b of the main article. Pearson correlation coefficients across 1,435 Mouse430_2-based microarray datasets were calculated among 778 ER genes. The gene list was based on a set downloaded from the AmiGO gene ontology database (GO:0005783) and modified by removing genes also associated with the Golgi (GO:0005794) or lysosomes (GO:0005764). The resulting data matrix (Additional file 2) was subjected to hierarchical clustering as described in Methods. The cluster with the largest average ($\bar{x}$ = 0.101), consisting of 97 ER genes (listed in Additional file 1), is highlighted in red. Click here for file

Additional file 4 **Genome-wide expression correlation analyses for mouse and human SREBF2 and XBP1.** (Worksheet 1, “Mouse”) Pearson correlations were averaged across the indicated number (n) of Mouse430_2-based datasets between the transcription factors Srebf2 or Xbp1 and 16,771 other named genes for which high-quality data were available. (Worksheet 2, “Human”) Pearson correlations between human SREBF2 or XBP1 and 17,236 other named genes averaged across the indicated number (n) of HG_U133_Plus2-based datasets. Click here for file

Additional file 5 **Transcription factors whose expression correlates with lysosomal genes.** Average expression correlations were calculated for each of 1412 human and 1066 mouse DNA-binding transcription factors across Affymetrix Mouse_430_2 and HG_U133_Plus2 arrays, respectively. We interrogated 1548 mouse and 1838 human datasets, but only those datasets with a minimum coefficient of variation of 5% for the respective reference gene were used for analyses (average number of datasets analyzed per gene, n=1061; range, 761-1281). In each case the 500 highest-ranking correlators were analyzed for GO term enrichment using a script based on the Bioconductor GOstats package [41]. The cutoff for p values was 0.001. The results were searched for the terms ‘lysosome’ or ‘vacuole’ and further filtered for genes encoding DNA-binding proteins that scored positive for both mouse and human data. GO term enrichment statistics are shown for the resulting 49 DNA-binding transcription factors. TF, transcription factor; ExpCount, expected number of genes in the selected 500-member list to be annotated with the respective GO term; Count, actual number of genes annotated with the GO term; Size, maximum number that could have been matched; Pvalue, hypergeometric probability; GO ID, gene ontology identifier. More detailed information is available at http://genecan.sgul.ac.uk. Click here for file

Additional file 6 **Matrix of expression correlations among lysosomal genes in mouse.** This supplement pertains to Figures 2 and 3. Data were generated as described in the accompanying legends. Tab-delimited. Click here for file

Additional file 7 **On each worksheet *Column A *** **lists lysosomal genes for the indicated cluster from Figure **2**.***Column C* lists NCBI GEO accession numbers of Mouse430_2 or HG_U133_Plus2-based gene expression data series that had been selected based on a minimum average coefficient of correlation among the reference genes of 30%. Values in *Column G* represent average Pearson correlation coefficients calculated between the corresponding transcription factor in *Column E* and the reference genes from *Column A* across the data series listed in *Column C. Column F* indicates whether the gene encodes a DNA-binding protein according to GO annotations (GO:0003677). Transcription factor genes are listed according to correlation values in descending order. Click here for file

Additional file 8 **Correlation of Stat6 with lysosomal genes.** (Worksheet 1, “Correl. Mouse”) Average expression correlations across 1,093 datasets were calculated between mouse *Stat6* and genes represented on Mouse_430_2 arrays as described in the legend to Additional file 4. (Worksheet 2, “Correl. Human”) Average expression correlations across 1,122 datasets were calculated between human *STAT6* and genes represented on HG_U133_Plus2 arrays as described in the legend to Additional file 4. (Worksheet 3, “GSEA Mouse”) The correlation-ranked gene list from Worksheet 1 was analyzed for GO term enrichment using the GSEA tool [57]. Gene set reference files in gene matrix transposed (gmt) format were generated by reformatting lists, downloaded from the AmiGO website, containing all mouse and human GO associations (all gene product types and data sources) [38]. Column J lists ‘leading-edge’ lysosomal genes as returned by GSEA; genes common to both the mouse and human leading edges are shown in red; leading-edge lysosomal genes bound by Stat6 according to reference [106] are boxed. (Worksheet 4, “GSEA Human”) The correlation-ranked gene list from Worksheet 2 was analyzed for GO term enrichment using the GSEA tool as in Worksheet 3. (Worksheet 5, “GOstats Mouse”) The 500 highest-ranking genes in Worksheet 1 were analyzed for GO term enrichment using a custom R script based on the Bioconductor GOstats package [41]. (Worksheet 6, “GOstats Human”) The 500 highest-ranking genes in Worksheet 2 were analyzed for GO term enrichment as in Worksheet 5. Click here for file

Additional file 9 **Effects of Stat6 and IL-4 on lysosomal gene expression in mouse bone marrow macrophages.** The data pertain to Figure 5 of the main article, and calculations were performed as detailed in the accompanying legend and in Methods. Values indicate log (base 2) fold changes of lysosomal genes as determined by gene expression profiling. Genes are color-coded according to GO annotations. Antigen processing and presentation, GO:0019882; glycosidases, GO:0016798; peptidases, GO:0008233; other hydrolases, GO:0016787; vesicular transport, GO:0016192; vacuolar H^+^ ATPase, GO:0016471. Click here for file

Additional file 10 **Stat6-bound loci are statistically enriched for genes encoding lysosomal proteins.** This analysis is based on publicly available ChIP-seq data as described in the legend to Figure 7. A file with peak coordinates in bed format was downloaded from the NCBI GEO depository [GEO:GSM1022305], and peaks were annotated as described in Methods. We only considered peaks whose centers were located within 5kb of transcription start sites of genes for which both HGNS gene symbols and Entrez gene IDs were available. The resulting list of 4,520 gene symbols was analyzed for GO set enrichment using the Bioconductor GOstats package [41]. The reference set consisted of 23,563 unique gene symbols based on a list of mouse GO annotations (version 1.79) from the Mouse Genome Information (MGI) site for which Entrez gene IDs were also available [122]. Click here for file

Additional file 11 **Stat6 binding to lysosomal loci verified by ChIP-PCR.** Bone marrow-derived macrophages from wild-type and Stat6-deficient mice were cultured in the presence of M-CSF for five days and then switched to media ± recombinant IL-4 for 30 minutes. Chromatin was crosslinked, fragmented and immunoprecipitated with control IgG (left panels) or anti-Stat6 (right panels) as described in Methods. The immunoprecipitated DNA was used as template for qPCR reactions to quantify selected Stat6 peak regions. Coordinates of PCR fragments are (mouse genome build mm9): Arg1, chr10:24650166-24650215; Plekhf1, chr7:39013036-39013202; Mmp13, chr9:7330552-7330598; Atp6v0d2, chr4:19821892-19821947; Atp6v0a1, chr11:100856194-100856250; Atp6v1b2, chr8:71615039-71615173; Ctsl, Chr13:64503469-64503600. Click here for file

Additional file 12 **Stat6 binds to active chromatin regions.** Analyses are based on publicly available ChIP-seq data deposited by Ostuni et al. [58]. Peak coordinates in bed format were downloaded from the NCBI GEO site [GEO:GSM1022256, GSM1022257, GSM1022258, GSM1022259, GSM1022260, GSM1022261, GSM1022262, GSM1022263, GSM1022264, GSM1022265, GSM1022266, GSM1022267, GSM1022297, GSM1022298, GSM1022299] and analyzed with the ChipPeakAnno package [112]. Columns A-C list the merged coordinates of 196 Stat6 peaks selected according to three criteria: (i) Stat6 peaks overlapped in all of three independent data sets obtained after growth of mouse macrophages with IL-4 for 1, 2 and 4 hours [GEO:GSM1022297, GSM1022298, GSM1022299]; (ii) peaks were filtered to exclude those located farther than 5kb from the nearest transcription start site; (iii) we further selected peaks close to genes annotated as encoding lysosomal proteins. Peak names in column E correspond to those in GSM1022299. Peaks were annotated with gene identifiers using information obtained through the BioMart server [123]. Columns H-S indicate whether the Stat6 peaks overlap with peaks obtained with antibodies against H3K26ac (columns H-K), H3K4me1 (columns L-O) or Pu.1 (columns P-S), using chromatin from wild-type or Stat6-deficient macrophages grown ± IL-4 for 4 hours as indicated in the column headers [58]. Click here for file

Additional file 13 **Oligonucleotides used for qPCR.** Primers were designed based on sequence information obtained through the NCBI Entrez Gene server [124] using the Primer-BLAST software tool [125]. Click here for file
