# Supplementary figures and images for: Reprogramming of lysosomal gene expression by interleukin-4 and Stat6
Source: BMC Genomics. 2013 Dec 5;14:853. doi: 10.1186/1471-2164-14-853 (PMC3880092; doi:10.1186/1471-2164-14-853)

### Additional file 3

(Brignull et al.)

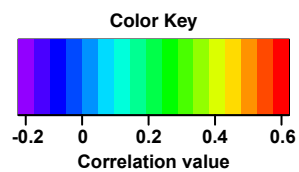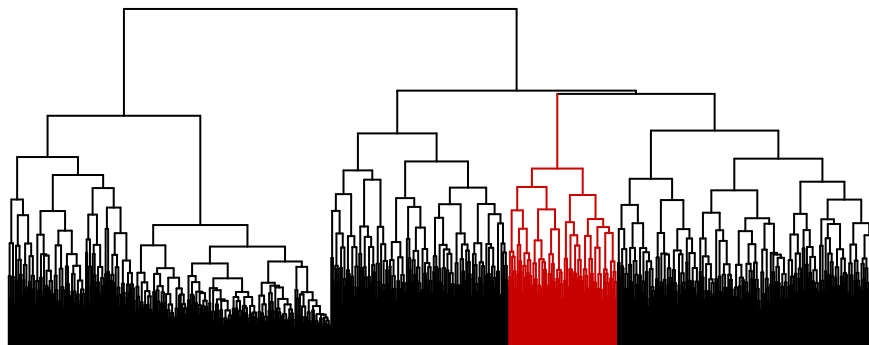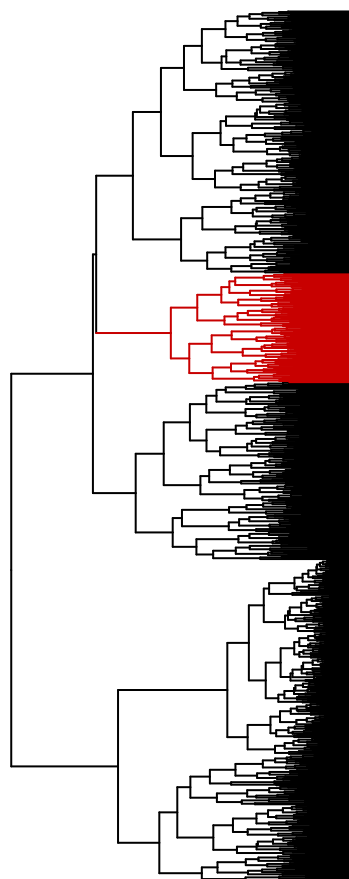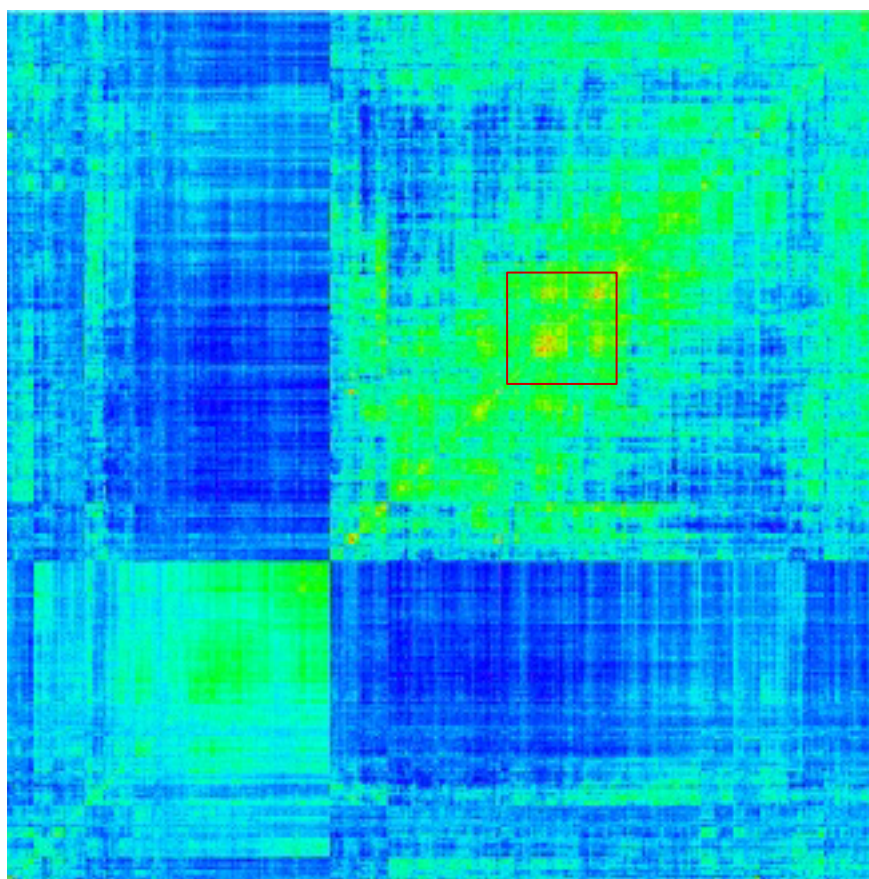

Supplement: Additional file 3 — Gene expression correlation analysis identifies distinct subsets of ER genes. These data pertain to Figure 1b of the main article. Pearson correlation coefficients across 1,435 Mouse430_2-based microarray datasets were calculated among 778 ER genes. The gene list was based on a set downloaded from the AmiGO gene ontology database (GO:0005783) and modified by removing genes also associated with the Golgi (GO:0005794) or lysosomes (GO:0005764). The resulting data matrix (Additional file 2) was subjected to hierarchical clustering as described in Methods. The cluster with the largest average (x¯ = 0.101), consisting of 97 ER genes (listed in Additional file 1), is highlighted in red. [file 1471-2164-14-853-S3.pdf]

Additional File 11  
(Brignull et al.)

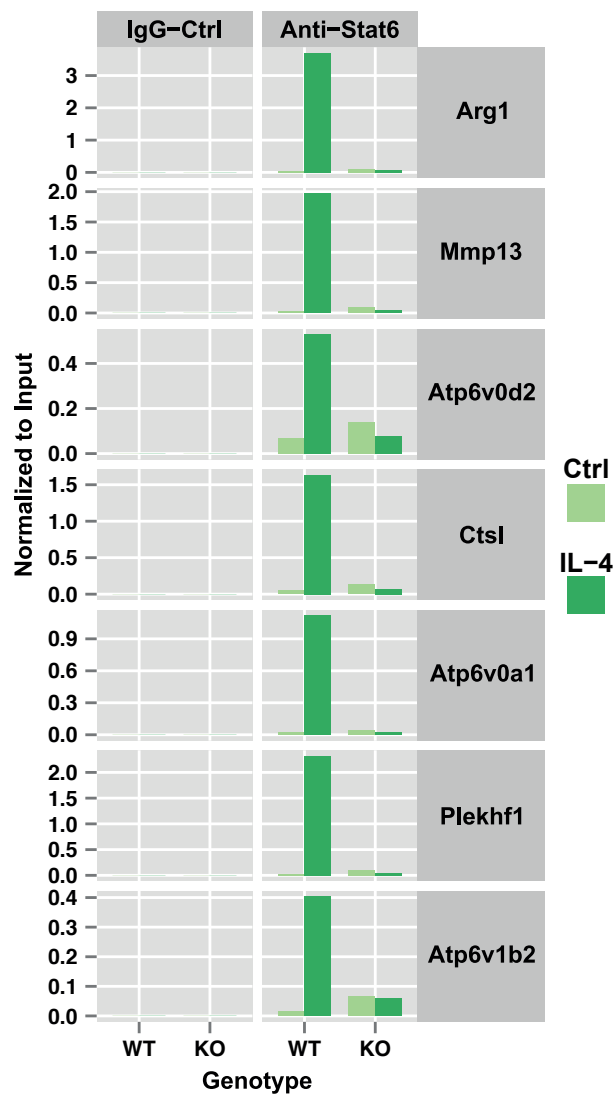

Supplement: Additional file 11 — Stat6 binding to lysosomal loci verified by ChIP-PCR. Bone marrow-derived macrophages from wild-type and Stat6-deficient mice were cultured in the presence of M-CSF for five days and then switched to media ± recombinant IL-4 for 30 minutes. Chromatin was crosslinked, fragmented and immunoprecipitated with control IgG (left panels) or anti-Stat6 (right panels) as described in Methods. The immunoprecipitated DNA was used as template for qPCR reactions to quantify selected Stat6 peak regions. Coordinates of PCR fragments are (mouse genome build mm9): Arg1, chr10:24650166-24650215; Plekhf1, chr7:39013036-39013202; Mmp13, chr9:7330552-7330598; Atp6v0d2, chr4:19821892-19821947; Atp6v0a1, chr11:100856194-100856250; Atp6v1b2, chr8:71615039-71615173; Ctsl, Chr13:64503469-64503600. [file 1471-2164-14-853-S11.pdf]
